# Emerging Infectious Diseases: Trends and Issues

**DOI:** 10.3201/eid0912.030558

**Published:** 2003-12

**Authors:** Elaine Larson

**Affiliations:** * Columbia University, New York, New York, USA

Preparing a text about emerging and reemerging infections sounds like a contradiction in terms since by the time a book is published, “new” infections may have come and gone. But Lashley and Durham have successfully walked the thin line between being dated on the one hand and providing timely, relevant data on the other. Several chapters place emerging infections and related problems such as microbial resistance in historical, cultural, and environmental context, which is relevant across diseases and time. The case study approach used for 17 specific diseases (e.g. cholera, cryptosporidiosis, malaria, prions, drug-resistant *Streptococcus pneumoniae*, West Nile virus) makes for an excellent vehicle for learning and fascinating reading. The book has five chapters on special issues—the role of infections in some cancers and chronic diseases, travel, immunocompromised persons, bioterrorism, behavioral and cultural aspects of transmission and infection—which cut across disease categories, as well as a future-looking summary. The book has four appendices for quick reference: emerging infections by organism and mode of transmission, prevention strategies, and a thorough list of resources. This book can serve as a valuable resource for epidemiologists, graduate students, and clinicians who need an overview reference text.

